# HMD-ARG: hierarchical multi-task deep learning for annotating antibiotic resistance genes

**DOI:** 10.1186/s40168-021-01002-3

**Published:** 2021-02-08

**Authors:** Yu Li, Zeling Xu, Wenkai Han, Huiluo Cao, Ramzan Umarov, Aixin Yan, Ming Fan, Huan Chen, Carlos M. Duarte, Lihua Li, Pak-Leung Ho, Xin Gao

**Affiliations:** 1 Computational Bioscience Research Center (CBRC), Computer, Electrical and Mathematical Sciences and Engineering (CEMSE) Division, King Abdullah University of Science and Technology (KAUST), Thuwal, 23955 Saudi Arabia; 2 Department of Computer Science and Engineering (CSE), The Chinese University of Hong Kong (CUHK), Hong Kong, People’s Republic of China; 3 School of Biological Sciences, The University of Hong Kong, Hong Kong, People’s Republic of China; 4 Carol Yu Center for Infection and Department of Microbiology, The University of Hong Kong, Hong Kong, People’s Republic of China; 5 Institute of Biomedical Engineering and Instrumentation, Hangzhou Dianzi University, Hangzhou, People’s Republic of China; 6 Key Laboratory of Microbial Technology and Bioinformatics of Zhejiang Province, Zhejiang Institute of Microbiology, Hangzhou, People’s Republic of China; 7 Biological and Environmental Sciences and Engineering (BESE) Division, King Abdullah University of Science and Technology (KAUST), Thuwal, 23955 Saudi Arabia

**Keywords:** Antibiotic resistance genes, Deep learning, Antibiotic class, Resistant mechanism, Gene mobility, Multi-task learning

## Abstract

**Background:**

The spread of antibiotic resistance has become one of the most urgent threats to global health, which is estimated to cause 700,000 deaths each year globally. Its surrogates, antibiotic resistance genes (ARGs), are highly transmittable between food, water, animal, and human to mitigate the efficacy of antibiotics. Accurately identifying ARGs is thus an indispensable step to understanding the ecology, and transmission of ARGs between environmental and human-associated reservoirs. Unfortunately, the previous computational methods for identifying ARGs are mostly based on sequence alignment, which cannot identify novel ARGs, and their applications are limited by currently incomplete knowledge about ARGs.

**Results:**

Here, we propose an end-to-end Hierarchical Multi-task Deep learning framework for ARG annotation (HMD-ARG). Taking raw sequence encoding as input, HMD-ARG can identify, without querying against existing sequence databases, multiple ARG properties simultaneously, including if the input protein sequence is an ARG, and if so, what antibiotic family it is resistant to, what resistant mechanism the ARG takes, and if the ARG is an intrinsic one or acquired one. In addition, if the predicted antibiotic family is beta-lactamase, HMD-ARG further predicts the subclass of beta-lactamase that the ARG is resistant to. Comprehensive experiments, including cross-fold validation, third-party dataset validation in human gut microbiota, wet-experimental functional validation, and structural investigation of predicted conserved sites, demonstrate not only the superior performance of our method over the state-of-art methods, but also the effectiveness and robustness of the proposed method.

**Conclusions:**

We propose a hierarchical multi-task method, HMD-ARG, which is based on deep learning and can provide detailed annotations of ARGs from three important aspects: resistant antibiotic class, resistant mechanism, and gene mobility. We believe that HMD-ARG can serve as a powerful tool to identify antibiotic resistance genes and, therefore mitigate their global threat. Our method and the constructed database are available at http://www.cbrc.kaust.edu.sa/HMDARG/.

Video abstract (MP4 50984 kb)

**Supplementary Information:**

The online version contains supplementary material available at 10.1186/s40168-021-01002-3.

## Background

The spread of antibiotic resistance has become one of the most pressing threats to global health, estimated to cause 700,000 deaths each year globally, with this number projected to increase to 10 million by 2050 if no action is taken [1, 2]. Antibiotic resistance genes (ARGs) are highly transmittable between food, water, animal, and human to mitigate the efficacy of antibiotics [3–7]. Accurately identifying ARGs is thus an indispensable step to understanding the ecology and transmission of ARGs between environmental and human-associated reservoirs [8].

Metagenomic high-throughput sequencing technologies have provided a quick and sensitive way to explore the ARGs in a single genome or metagenomic samples, and many bioinformatic tools were proposed to annotate genes from the metagenomic datasets. Most methods fall into two categories: assembly-based methods, those that assemble sequencing reads into contiguous fragments and then search against reference databases, and the read-based methods, those that align raw reads to reference alleles directly [9]. The widely used AMRPlusPlus [10] is a typical example of the read-based methods. It directly aligns short reads to a custom reference database using BWA [11] to predict the presence of ARGs. Such methods scale well for complex metagenomic samples, but they may cause a large number of false positives due to local sequence similarity. On the other hand, the assembly-based methods require assemblers like SPAdes [12], Velvet [13], IDBA-UD [14], MEGAHIT [15], and then comparing predicted protein-coding regions against reference databases to identify ARGs. The assembler’s performance may influence the overall ARG prediction, but the upstream and downstream positional information from the assembly can compensate for the false-positive problem to some extent. The difference between these two kinds of approaches is in the pre-processing step, whereas their core ideas are similar. That is, they use the pairwise alignment or multi-sequence alignment algorithms to identify and annotate resistance genes.

As discussed above, the alignment-dependent methods are widely used, but they have the following disadvantages. Firstly, due to the pairwise alignment setting, the assembly-based methods are not sensitive to point mutations. Consequently, they may ignore novel ARGs and have limited power in mechanism analysis. Secondly, if the users want to obtain satisfactory results using those methods, they should have a clear understanding of how to set the parameters in those algorithms correctly, such as the similarity threshold. Such a domain knowledge requirement limits the real usage of the methods, which is one of the reasons that many people find the existing tools not that useful. Thirdly, because the alignment-based methods depend on the curated databases, such methods cannot identify novel ARGs, and their applications are limited by currently incomplete knowledge about ARGs [16]. Machine learning methods can potentially learn the statistical patterns of ARGs and be able to predict novel ones [17–20]. In particular, deep learning may circumvent these obstacles because of its intrinsic superiority in feature extraction from raw data. Recently, building upon a multi-layer perceptron model, DeepARG [21] was developed to identity ARGs using similarity features by comparing the query sequence to the existing ARG databases. Although similarity might extract effective features to identify ARGs, DeepARG still inherits the disadvantage of alignment-based methods. In addition, existing methods cannot predict the gene mobility, that is, whether the ARG is intrinsic [22] or could be acquired [23] via horizontal gene transfer [24].

**Fig. 1:**
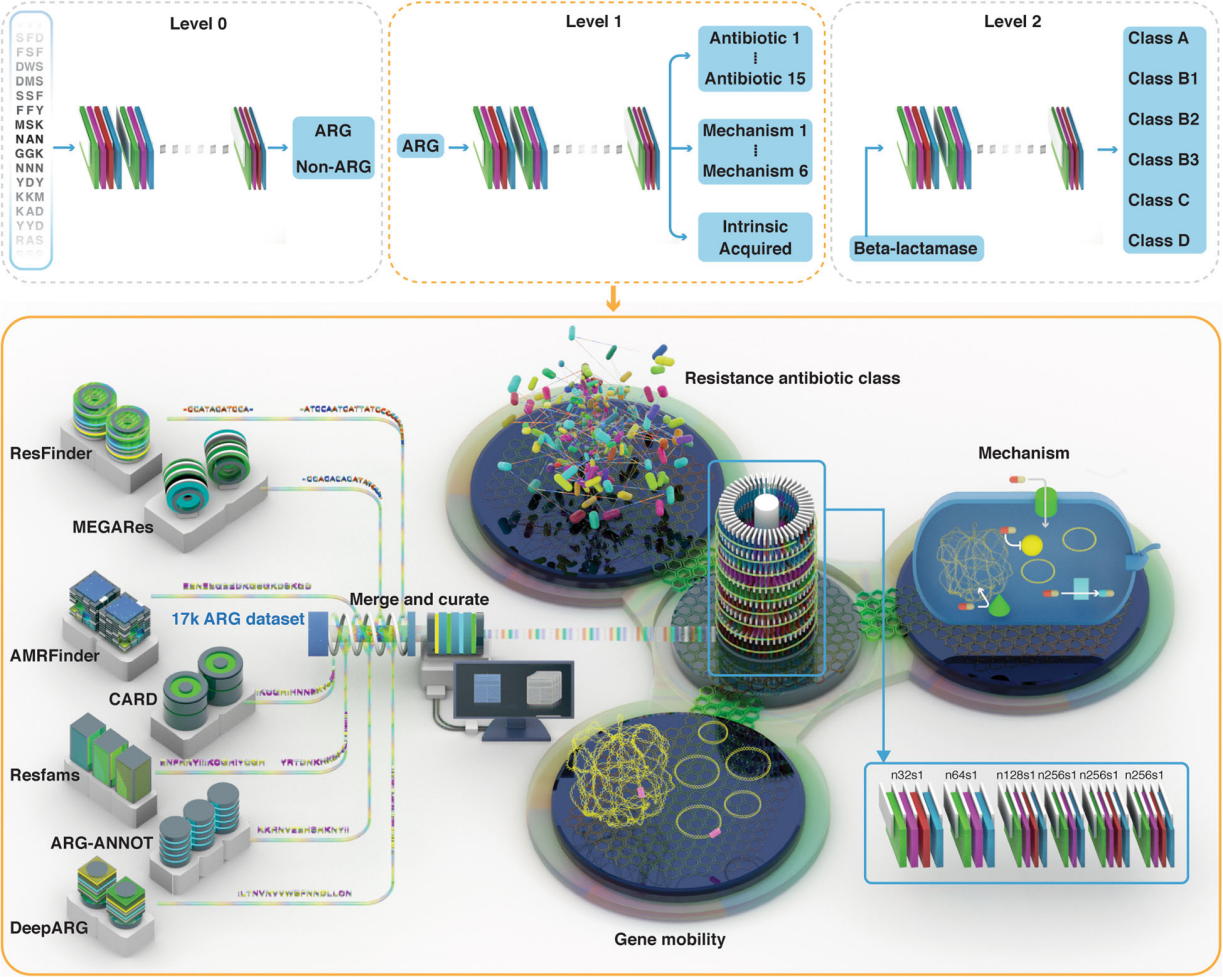
Overview of HMD-ARG. Top panel: HMD-ARG is composed of three deep learning models, which are responsible for three level predictions. In level 0, one model is trained to predict whether an input sequence is an ARG or not. If it is an ARG, it will go through the second level prediction, in which a multi-task deep learning model (more details shown in the bottom panel) is trained to predict the resistant antibiotic family, resistant mechanism, and gene mobility information at the same time. If the sequence is predicted as beta-lactamase in level 1, it will be fed into the level 2 model to predict its beta-lactamase subclass. Bottom panel: In order to train those models, we built the most comprehensive ARG database to date by merging the sequences from seven existing databases, followed by a post-processing step to remove duplicates. Then, we used the existing tools and manual curation to assign the annotations from three aspects, i.e., resistant antibiotic family, resistant mechanism, and gene mobility, to each sequence in the database. Those sequences were then fed to deep learning models to train our models, as illustrated in the right part

In this work, we propose a multi-task deep learning framework, called HMD-ARG (Fig. 1). With the raw sequence encoding as input and without querying against existing sequence databases, HMD-ARG predicts multiple ARG properties simultaneously, including resolving if the input protein sequence is an ARG, and if so, what antibiotic family it is resistant to, what resistant mechanism is involved in the ARG, and whether the ARG is intrinsic or acquired. In addition, if the predicted antibiotic family is beta-lactamase, HMD-ARG further predicts the subclass of beta-lactamase that the ARG is resistant to.

## Methods

### Database description

**Fig. 2:**
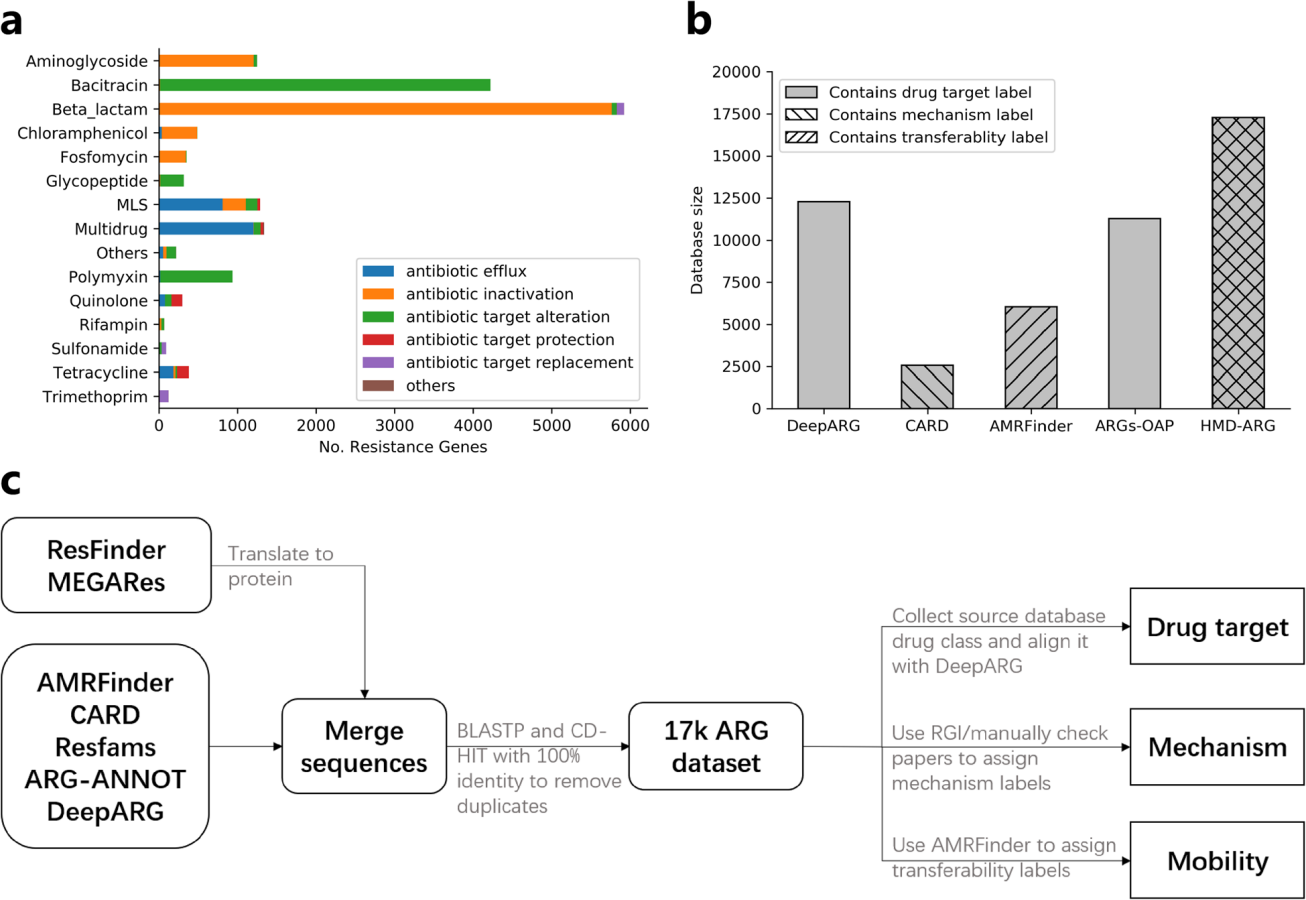
HMD-ARG database composition and the HMD-ARG database construction pipeline. **a** The statistics of the HMD-ARG database. The number of sequences belonging to each antibiotic family is different. Meanwhile, different genes can resist the same drug with different mechanisms, which are shown in different colors. **b** Different databases have various numbers of sequences as well as different labeling information. HMD-ARG database is currently the largest one. At the same time, it is the most comprehensive one, with resistant antibiotic class, resistant mechanism, and gene mobility labeled. **c** To construct the database, we merged the sequences from seven existing databases, followed by a post-processing step to remove duplicates. Then, we either used the existing tools or manual curation to assign the annotations from three aspects to each sequence in the database

We curated a comprehensive multi-label ARG database, HMD-ARG-DB, with a high degree of confidence and extensively manual curations, which can serve as a valuable resource for the community. We collected and cleaned resistance gene sequences from seven published ARG databases: Comprehensive Antibiotic Resistance Database (CARD) [25], AMRFinder [26], ResFinder [27], Antibiotic Resistance Gene-ANNOTation (ARG-ANNOT) [28], DeepARG [21], MEGARes [10], and Resfams [8]. Then, we labeled these sequences from three perspectives with manual check (Figs. 1 and 2c), (1) the antibiotic class they confer to, (2) the mechanism of antibiotic resistance, (3) and transferable ability. We removed the identical and duplicate sequences from our database following the same procedure as DeepARG [21].

The resulting database, HMD-ARG-DB, is composed of 17,282 high-quality sequences, coupled with labels of 15 antibiotic classes, 6 underlying resistance mechanisms, and their mobility (Database construction in Supp, Fig. 2a). This type of multiple label database ensures the trained models to capture the most relevant features associated with ARGs automatically. Over 30 % of the genes belong to beta-lactam category (5921), most of which perform the resistance function through inactivating beta-lactams (5763). About 24% of the genes are assigned to bacitracin category (4219), and almost all of them perform antibiotic target alternation (4206) (Fig. 2a). HMD-ARG-DB is the largest database to date, including the most comprehensive annotations on the three different aspects targeted (Fig. 2b).

### Overview of HMD-ARG

HMD-ARG is a supervised machine learning framework for ARG annotation, consisting of three models (Fig. 1 top panel), with a level-by-level prediction strategy. Given a protein sequence, HMD-ARG can annotate it from the following three aspects: antibiotic resistance type, mechanism, and gene mobility. More specifically, regarding the antibiotic resistance type, HMD-ARG predicts which of the 15 antibiotic families the predicted ARG is resistant to. As for the mechanism, HMD-ARG annotates the ARG based on the biochemical basis of its resistance, including antibiotic efflux, antibiotic inactivation, antibiotic target alternation, antibiotic target protection, antibiotic replacement, and others. In terms of gene mobility, HMD-ARG distinguishes the intrinsic genes from the acquired ones in plasmid.

To perform these predictions, HMD-ARG is designed to have three models with a level-by-level prediction strategy, which deploys the hierarchical structure of the ARG labeling space. For a given protein sequence, it first classifies the sequence into ARG or non-ARG; if the input sequence is an ARG, we predict its resistant antibiotic type; if the ARG is a beta-lactamase, we will further annotate it with the refined beta-lactamase subtype. Accordingly, given any sequence analyzed by the HMD-ARG framework, the first model (level 0) predicts whether it is ARG or non-ARG. If it is an ARG, the second multi-task model (level 1) predicts the resistant antibiotic type, the underlying mechanism of resistance, and gene mobility. Furthermore, if the ARG is predicted to resist to beta-lactam, the third model (level 2) predicts its molecular subclass [29]. This hierarchical framework helps deal with data imbalance problem and reduces the computational complexity for non-ARG.

For each level of the prediction, the model is based on an end-to-end convolutional neural network (CNN) model, taking the raw representation of the sequence, i.e., one-hot encoding, as inputs. To increase the capacity, the model used in level 1 contains one additional layer before the final multi-task outputs. The structure of the multi-task learning model is illustrated in Fig. 1 bottom panel. More details of the model structure could be referred to the “Deep learning model” and “Implementation details” sections below.

### Deep learning model

In each level of the prediction, the model is an end-to-end convolutional neural network (CNN) model. For these models, the inputs are protein sequences, which are strings composed of 23 characters representing different amino acids. To render the inputs suitable for the deep learning mathematical model, we used one-hot encoding to represent the input sequences. Then, the sequence encodings go through six convolutional layers and four pooling layers, which are designed to detect important motifs and aggregate both useful local and global information across the entire input sequence. The outputs of the last pooling layer are flattened and then fed into three fully-connected layers, which are designed to learn the functional mapping between the learned representation from the convolutional layers and the final labeling space. Since all of our tasks are classification ones, for the ARG/non-ARG and the beta-lactam subtype prediction, we used the standard cross-entropy loss. The multi-task learning loss function will be discussed in “Multi-task learning” section below. More details about the hyper-parameter setting are discussed in the “Implementation details” section and “Model hype-parameters” in Supp.

### Multi-task learning

Within the HMD-ARG framework, there is one model (level 1) performing multi-task learning for the coarse resistant antibiotic type, functional mechanism, and gene mobility prediction. The model architecture is roughly the same as that described in the “Deep learning model” section above. However, for the last layer of the model, instead of only having one fully-connected branch with a Softmax activation function, we have three fully-connected branches, which correspond to the three tasks, respectively. In other words, the model for multi-task learning is essentially composed of three models, while those models share the convolutional and pooling layers. One clear advantage of this multi-task learning framework is that the three tasks altogether force those layers to discover important features within the input sequences, which are useful for all the three tasks, and thus prevent the model from overfitting. On the other hand, the loss function is changed accordingly:

$$ {L}_{\mathrm{multi}-\mathrm{task}}=\alpha \ast {L}_{\mathrm{drug}}+\beta \ast {L}_{\mathrm{mechanism}}+\gamma \ast {L}_{\mathrm{source}}, $$

where *α*, *β*, *γ* are the weights of the three tasks and are hyper-parameters; *L*_drug_, *L*_mechanism_, and *L*_source_ are the cross-entropy losses of the corresponding tasks, respectively. Essentially, we optimize over the weighted *L*_multi − task_, instead of each cross-entropy loss alone, to take care of all the three tasks simultaneously. After training the above model, given an input sequence, we obtain the prediction results of the three tasks with one single forward-propagation.

### Implementation details

We collected 66k non-ARGs from UniProt [30] with highest BLAST similarity scores against the ARGs in HMD-ARG-DB, used them as the negative set so that the negative set is as similar to the positive one as possible to force HMD-ARG to learn a more powerful model, and then trained the level 0 model on the combined dataset. The level 1 multi-task learning was performed with HMD-ARG-DB. For beta-lactamase subclass prediction, we trained our model on an up-to-date beta-lactamase database, Beta-Lactamase DataBase [31] (BLDB). The database contains more than 4000 beta-lactamases sequences. Each of them has a molecular class label, indicating which subclass the sequence belongs to. In total, there are 6 subclasses, class A, B1, B2, B3, C, and D.

When training the models, we first converted each amino acid into a one-hot encoding vector. So, protein sequences are converted into a zero-padded numerical matrix with the dimension as 1576 by 23, where 1576 meets the length of the longest ARGs and non-ARGs in our dataset, and 23 stands for 20 standard amino acids, two infrequent amino acids (B, Z), and X for unknown ones. Such an encoded matrix is then fed into a deep learning model with six convolutional layers and four max-pooling layers. The model hyper-parameters are discussed in “Model hype-parameters” in Supp.

### Saliency map construction

The saliency map is constructed as follows. At each position of the protein sequence, we replaced the amino acid with a different amino acid, obtaining a mutated sequence, and fed the mutated sequence to the level 0 model, which outputs the predicted probability of the mutated sequence being an ARG. We performed this procedure for each amino acid replacement and each position. As a result, the saliency map has a dimension of *L* by 20, where *L* is the length of the sequence. When constructing the average saliency map, we first performed multiple sequence alignment for a specific sequence against our database, obtaining the alignment results. Then, we built a saliency map for each sequence that can be aligned to the query sequence. Finally, we aligned the saliency map based on the sequence alignment results and took the average of the maps to obtain the average saliency map. To compare our method against the sequence-alignment based methods, we built a position-specific scoring matrix [32] (PSSM) with PSI-BLAST [33], visualized the PSSM, and compared it with our saliency map.

### Wet experimental expression of predicted ARGs

Eight predicted ARGs in *Pseudomonas aeruginosa* strain PA150567 were selected for this purpose. The ORF regions of the predicted genes were amplified by PCR using the iProof™ High-Fidelity DNA Polymerase (Bio-Rad, USA) with the primers as listed in Table S2. Purified DNA fragments and the pET28a vector were digested with *BamH*I and *Xho*I (NEB, USA). After ligation using the Quick Ligation^TM^ Kit (NEB, USA) and verification by PCR and DNA sequencing (BGI, China), the resulting plasmids were transformed into *E. coli* BL21 for antibiotic sensitivity analysis. Overnight culture of *E. coli* BL21 strains containing the plasmids for overexpression of the predicted genes were 1:100 diluted and grown in LB medium supplemented with kanamycin (20 μg/ml) and Isopropyl β-D-1-thiogalactopyranoside (IPTG) (0.5 mM). After incubation at 37 °C with 220-rpm agitation for 90 min, bacterial cultures were transferred to a 24-well plate. After antibiotic was added with indicated concentration (ampicillin: 50 μg/ml, carbenicillin: 10 μg/ml; meropenem: 2 μg/ml; amikacin: 4 μg/ml), cell growth (OD600nm) was measured every 10 min at 37 °C in the Synergy HTX Plate Reader (BioTek, USA) with agitation. Assays were performed in duplicate.

## Results

### Overall performance of HMD-ARG

We first used 5-fold stratified cross-validation to evaluate the performance of HMD-ARG and compared it with the state-of-the-art methods [21, 25, 34, 35]. In this experiment, we randomly divided HMD-ARG-DB into five folds. Each time, we chose four folds of the dataset for the model training and tested the trained model on the remaining one. To avoid data bias, average results were generated from repeating the above procedure for five times. In general, as shown in Tables 1, 2, 3, 4, and 5, HMD-ARG achieves the state-of-the-art results on all the tasks (evaluation criteria definitions and the detailed steps of executing other methods provided in “Evaluation criteria” and “Protocols of other methods” in Supp). More specifically, for the most important task, antibiotic class prediction (Table 2), HMD-ARG shows significantly higher accuracy (0.935), recall (0.847), and F1 score (0.893) with a promising precision (0.950) than the existing methods. It is not surprising that CARD has a slightly better precision score (0.981), because the database was curated based on sequence similarity search. However, similarity-based methods produce more false negatives, resulting in a low recall rate, e.g., in CARD (0.452), AMRPlusPlus (0.278), and Meta-MARC (0.782). By combining similarity features with a multi-layer perception, DeepARG improves the overall prediction results, compared with CARD, with the precision, recall, and F1 score being 0.914, 0.757, and 0.814, respectively, but it is still much lower than HMD-ARG. For the resistance mechanism prediction (Table 3), HMD-ARG significantly outperforms CARD, especially on recall. As for the gene mobility prediction (Table 4), our model can also achieve impressive results, while the other commonly used methods cannot perform this task. Although HMD-ARG is not designed specifically for beta-lactamase prediction (Table 5), our method still achieves remarkably accurate results, which is similar to the state-of-the-art method that is trained specifically on this task, CNN-BLPred [36].

**Table 1:** The ARG/non-ARG classification results between different methods

	Accuracy	Precision^a^	Recall	F1-score
HMD-ARG	0.948	0.939	**0.971**	0.948
CARD	0.71	**0.999**	0.421	0.592
DeepARG	**0.965**	0.998	0.93	**0.963**
AMRPlusPlus^b^	0.691	0.867	0.449	0.592
Meta-MARC^c^	0.848	0.847	0.85	0.848

^a^The precision, recall, and F1-score are only for ARG

^b^AMR++ requires the input in a paired fastq format. So, we simulated the fastq data from our test protein dataset. Details can be seen in the supplementary

^c^Meta-MARC can work with both assembly and raw data; we tested it with the assembled sequences (our test dataset)

**Table 2:** The ARG antibiotic classes’ classification results between different methods

	Accuracy	Precision^a^	Recall	F1-score
HMD-ARG	**0.935**	0.950	**0.847**	**0.893**
CARD	0.418	**0.983**	0.452	0.585
DeepARG	0.887	0.914	0.757	0.814
AMRPlusPlus^b^	0.675	0.574	0.278	0.283
Meta-MARC^c^	0.909	0.750	0.782	0.745

^a^The precision, recall, and F1-score are the macro average over the ARG antibiotic classes

^b^AMR++ requires the input in a paired fastq format. So, we simulated the fastq data from our test protein dataset. Details can be seen in the supplementary

^c^The drug class label system of AMR++ and Meta-MARC are different from ours, so we manually converted their labels into ours. Meta-MARC can work with both assembly and raw data; we tested it with the assembled sequences (our test dataset)

**Table 3:** The ARG antibiotic mechanism classification results between different methods

	Accuracy	Precision^a^	Recall	F1-score
HMD-ARG	**0.936**	**0.867**	**0.768**	**0.795**
CARD	0.423	0.832	0.476	0.566

^a^The precision, recall, and f1-score are the macro average over the ARG mechanism classes

**Table 4:** The ARG antibiotic mobility classification results

	Accuracy	Precision^a^	Recall	F1-score
HMD-ARG	0.909	0.964	0.89	0.926

^a^The precision, recall, and F1-score are the macro average over the ARG mobility classes

**Table 5:** The beta-lactamases Ambler classification results between different methods

	Accuracy	Precision^a^	Recall	F1-score
HMD-ARG	**0.995**	**0.989**	**0.993**	**0.991**
CNN-Blpred	0.987	0.983	0.991	0.986

^a^The precision, recall, and F1-score are the macro average over the beta-lactamases molecular type classes

### Robustness of HMD-ARG

**Fig. 3:**
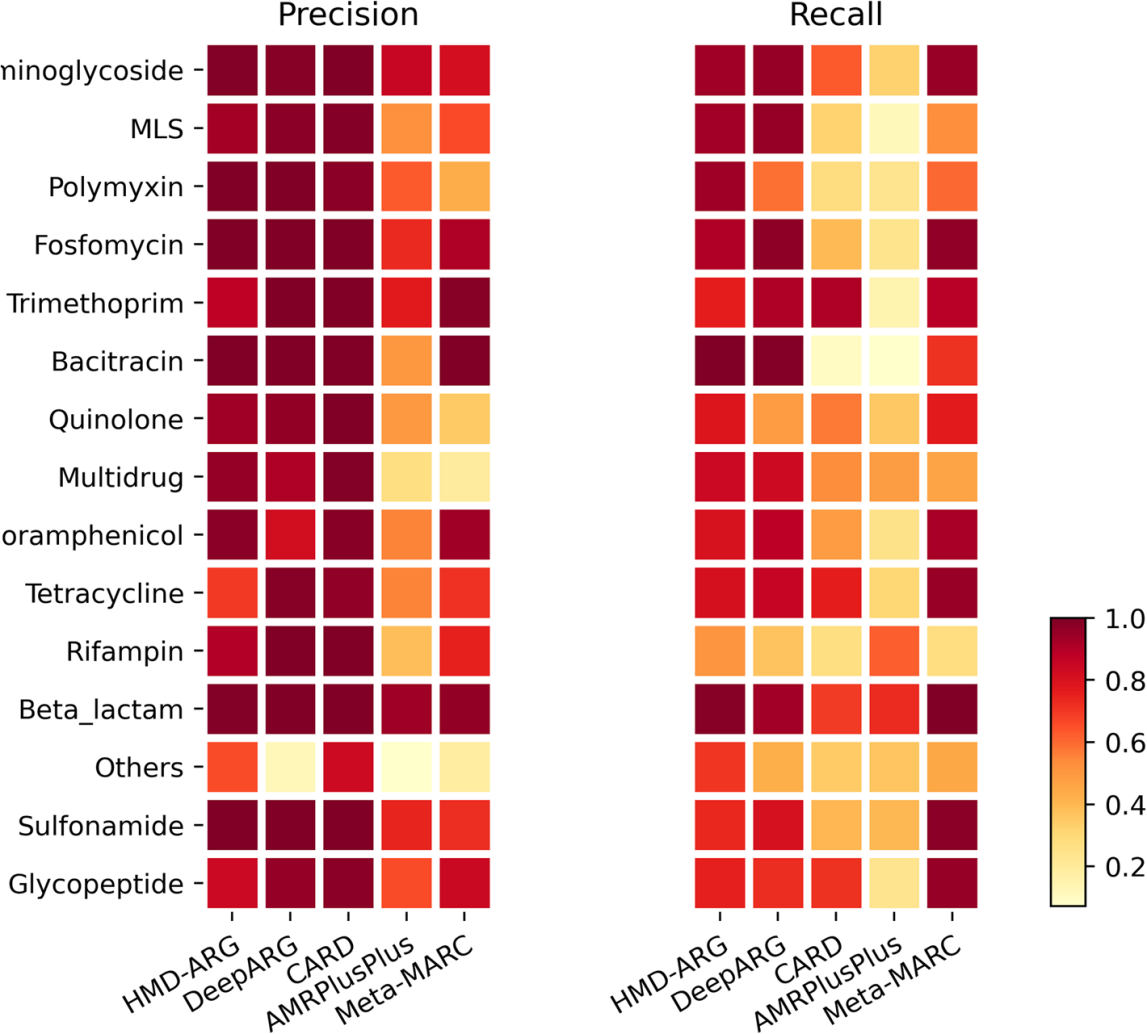
Detailed prediction performance comparison. Detailed performance comparison of HMD-ARG, DeepARG, CARD, AMRPlusPlus, and Meta-MARC on each resistant antibiotic class. Meta-MARC can work with both assembly and raw data; we tested it with the assembled sequences

We further investigated the robustness of HMD-ARG, especially on the ARG/non-ARG prediction and antibiotic class prediction. HMD-ARG appears to be outperformed by DeepARG in discriminating ARGs from non-ARGs (Table 1). DeepARG’s precision on identifying ARG is very high, but this should be attributed to its sequence similarity filtering process before feeding their model. There is no doubt that the pre-filtering used in DeepARG has inherited the disadvantage of similarity-based methods, resulting in a high false negative rate and thus low recall. Furthermore, the similarity threshold prerequisite might diminish the probabilistic model used in DeepARG, eventually attenuating the robustness of the method. On the contrary, taking the sequence one-hot encoding as input, without additional pre-processing, HMD-ARG remains as a genuine probabilistic model. This might explain the better robustness of HMD-ARG than DeepARG referring to the ROC curve (Fig. S1). The ROC curve shows the smoothness in HMD-ARG’s prediction in contrast to a sharp changing point that is caused by the similarity threshold in DeepARG. Moreover, the area under ROC curve (AUROC) of HMD-ARG (0.99) is higher than that of DeepARG (0.97). Regarding the performance on each antibiotic class, HMD-ARG is quite stable across different classes, regardless of precision or recall (Fig. 3). On the other hand, DeepARG and CARD have a very diverse performance on different classes, especially in terms of recall. The robustness of HMD-ARG, on different levels and different classes, indicates that it is a reliable tool.

### Predicting novel ARGs in human gut microbiota

Identification of ARGs in human gut metagenomes has a high clinical significance, because a large portion of bacteria are uncultured [37, 38]. The resistome in the human gut is also expected to be quite different from ARGs in current databases since most known ARGs are from cultured pathogens [37, 38]. Evaluating the proposed method on the new third-party human gut dataset shows the ability of HMD-ARG in identifying novel ARGs. We collected the human gut microbiota dataset, Mustard database, which contains 6095 predicted ARGs in 20 families, from the most recent work [39]. These labeled ARGs were predicted from a 3.9 million human gut microbiome gene catalog, using a three-dimensional structure-based method, with a portion of the sequences being experimentally verified. Then, we compared the sequences in the Mustard database with the training sequences for HMD-ARG, ensuring that there is no overlap between the two. Finally, we applied HMD-ARG and DeepARG onto this third-party dataset. The numbers of novel ARGs belonging to different classes correctly recovered by HMD-ARG and DeepARG were used as surrogate measures for the prediction performance. Although DeepARG has better performance on two classes, HMD-ARG outperforms it in 15 out of the 20 classes, being able to identify more novel ARGs (Fig. S2). Moreover, although HMD-ARG only utilizes sequence information, it can even achieve similar performance as the structured-based method on some classes. Compared with the structured-based method, HMD-ARG has a broader application scenario since it does not require structural information as the input.

### Experimental validation

**Fig. 4:**
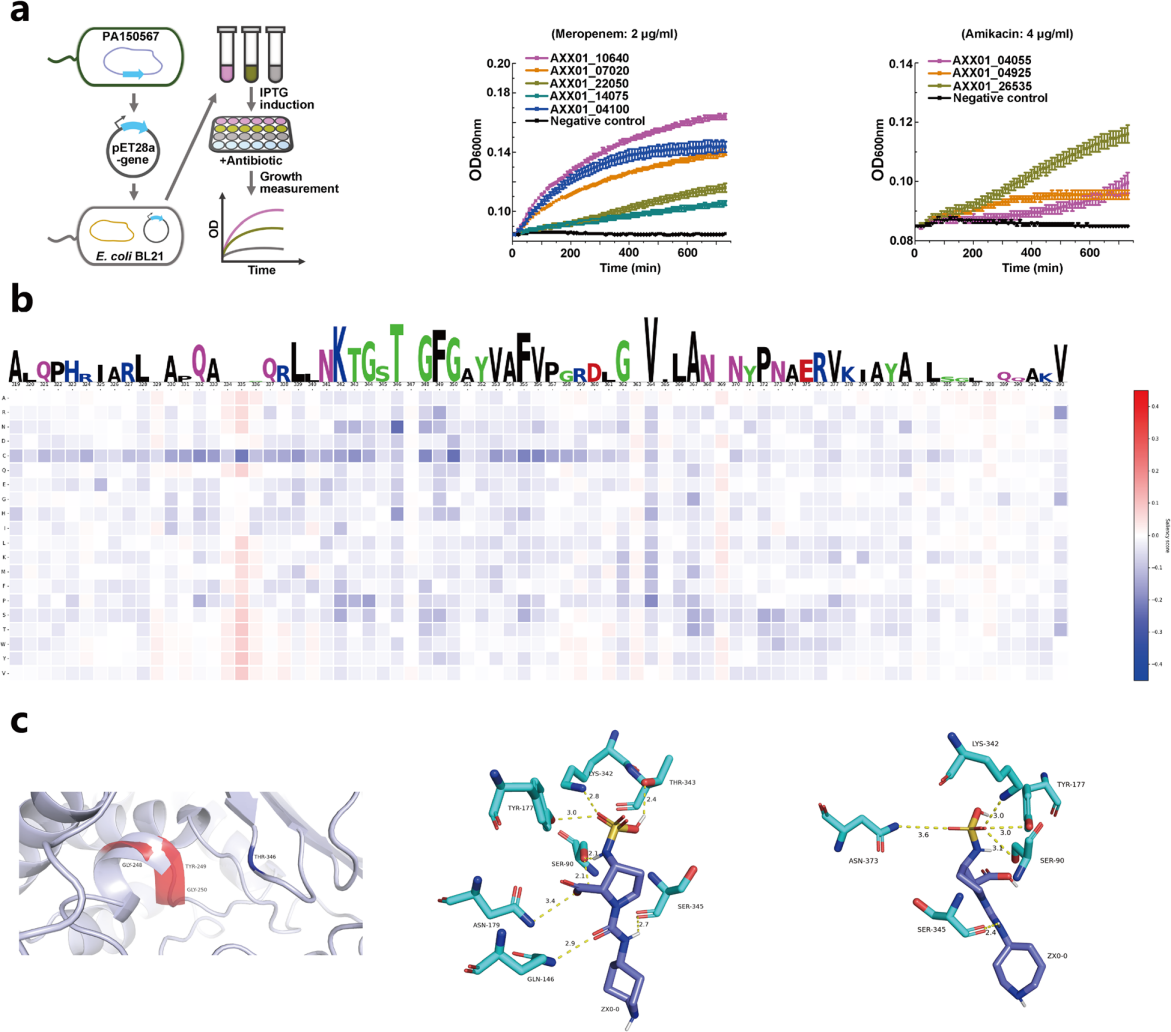
Functional validation of the predicted ARGs and structural investigation of conserved sites detected by HMD-ARG. **a** Left figure: A diagram showing the procedure of heterologous expression and functional analysis of the predicted ARGs from PA150567 in *E. coli* BL21 host. Middle figure**:** Growth curves of *E. coli* hosts with expression of the predicted *β*-lactamases that inactivate *β*-lactam antibiotics in the presence of 2 μg/ml meropenem. Right figure**:** Growth curves of *E. coli* hosts with expression of the predicted acetyltransferases that inactivate aminoglycoside antibiotics in the presence of 4 μg/ml amikacin. **b** The HMD-ARG predicted conserved sites and the corresponding sequence logo from 319 to 393 in AXX01_04100. **c** After we mutated the conserved site (346) from T to N, the mutated protein’s (colored in red) local structure (predicted by RaptorX) changed significantly from the wild type (colored in light blue). Moreover, the binding affinity (predicted by AutoDock) between the mutated protein and the ligand (antibiotics) also reduced, as illustrated in the middle (wild type) and right (mutated protein) figures

To validate the performance of our model on predicting ARGs, five genes assigned to beta-lactamase and three genes predicted as the aminoglycode class from a clinical strain *Pseudomonas aeruginosa* PA150567 were randomly selected for wet lab experimental validation (Table S1 and Fig. 4a). Note that some of those randomly selected genes have a BLAST [40] identity against the database lower than 50%, which means they cannot be detected by the similarity-based methods, including DeepARG. All of these genes were heterologously expressed in *Escherichia coli* BL21 host (Fig. 4a left). The growth was measured in presence of meropenem (Fig. 4a middle) and amikacin (Fig. 4a right) for each successful transformant with corresponding ARGs predicted in the present study. Results demonstrate the antibiotic resistance of all of these ARGs in *E. coli* transformants and confirm the good performance of our model. Although the lower activities of predicted ARGs to two beta-lactam antibiotics were observed than the one showing 100% identity with known ARGs (Fig. S3), the activities were still higher than those without transformed ARGs. The lower functions are possibly attributed to the unclear regulatory systems in vivo or the genuine low enzyme activities of these predicted ARGs. Altogether, since we randomly selected these genes for validation, this might be applicable for all predicted ARGs, at least for those with mechanisms underlying inactivating antibiotics.

### Capturing evolutionary information

To further investigate the improved performance of HMD-ARG, we first performed a saliency map (mutation map) analysis [41] on the HMD-ARG model. For each amino acid in an ARG sequence, we mutated it to a different amino acid and fed the mutated sequence to the trained HMD-ARG, obtaining the predicted probability of the mutated sequence being an ARG. Since each amino acid can be mutated to 20 amino acids (including itself), the saliency map for each ARG will have a dimension of *L* by 20, where *L* is the sequence length. From this mutation map analysis, we were able to obtain the conserved sites predicted by our model (Fig. 4b). Taking a beta-lactamase resistance gene, AFB78806 [42] as an example, we built the saliency map for every sequence in HMD-ARG-DB that could be aligned to it. Then, based on the sequence alignment, we aligned the saliency map and obtained the averaged saliency map for the AFB78806 group. Meanwhile, we built the position-specific scoring matrix (PSSM) for AFB78806. The averaged saliency map and PSSM are shown in Fig. S4. The overall Pearson correlation coefficient as 0.24 between the averaged saliency map and PSSM was observed. Particularly, the most distinct positions, such as column 226, were determined with a Pearson correlation coefficient higher than 0.5. PSSM can capture the evolutionary information for a specific sequence, with a high value in the matrix indicating a highly conserved position. On the other hand, the physical meaning of the averaged saliency map is that if there is a mutation with high resistant probability, this specific mutated gene is likely to survive. Considering that when building the HMD-ARG models, we only used the raw sequence information, without utilizing the evolutionary information or the alignment information, the correlation between the group saliency map and the PSSM indicates two things. Firstly, the prediction of HMD-ARG is robust and consistent within a group of ARGs; otherwise, the averaged saliency map would not show clear patterns. Secondly, HMD-ARG is able to detect the evolutionary information in an implicit way, capturing the conserved positions and mutations that are critical to the activity of enzymes. Thus HMD-ARG may serve as a tool for analyzing the evolution of ARGs.

### Predicted conserved sites validation

We validated the predicted conserved sites obtained from the model analysis above. We used the protein AXX01_04100, which was validated by bioassays, as an example. After performing the mutation map analysis, we obtained the conserved site sequence logo shown in Fig. 4b. As illustrated in the figure, we could recover some known conserved sequence motifs, such as KTG [43] (342–344). For comparison, we also built a PSSM to check the ability of multiple sequence alignment-based methods in capturing the motifs. The results are shown in Fig. S5. We found that the PSSM is powerful in capturing evolutionally conserved amino acids, but the KTG signal in the figure is not so clear compared with that in the saliency map of our method. In addition, we wanted to validate the previously unknown conserved sites, which were predicted by our analysis. We chose the most significant signal from the mutation map, i.e., 346 (T to N), and made such mutation on the protein sequence. Using RaptorX [44], we obtained the predicted structure of the mutated protein, which was then aligned and compared with the wild type protein structure. The comparison results were illustrated in Fig. 4c left figure. Although we only mutated one single amino acid, the local structure and environment have changed significantly, which indicates the importance of such a conserved site. To further test the newly predicted conserved site, we performed docking experiments with AutoDock Vina [45], using the beta-lactam antibiotic, to bind against both the wild type structure and the mutated one. As shown in Fig. 4c middle (wild) and right (mutated) figures, the binding affinity between the protein and the antibiotics has indeed decreased significantly from the wild type (− 7.8 kcal/mol) to the mutated protein (− 6.8 kcal/mol), although these in silico analyses demonstrate the applicability of HMD-ARG in recognizing the antibiotic conserved sites and motifs.

## Discussion and conclusions

We developed a hierarchical multi-task method, HMD-ARG, based on deep learning, to facilitate the detection and understanding of antibiotic resistance, providing detailed annotations of ARGs from three important aspects: resistant antibiotic class, resistant mechanism, and gene mobility. Comprehensive experiments, including cross-fold validation, third-party dataset validation, wet-experimental functional validation, and structural investigation of predicted conserved sites, demonstrate the effectiveness and robustness of the proposed method.

Most known ARGs were found from culturable bacteria, and people used sequence comparison to explore and identify those sequences. As a result, tools based on sequence alignment, like DeepARG and CARD, could achieve satisfactory precision. However, the performance of DeepARG on characterizing the ARGs from intestinal microbiota (which are generally not cultured and distantly related to known ARGs) is worse than that of our deep learning model, although both methods are trained on known ARGs from culturable bacteria. Indeed, currently, there is no consensus on the optimal approach to detect ARGs, and all the methods play the trade-off between precision and recall. Very recently, DeepARG pushed a new version. We also did the cross-validation experiments with the latest version, finding out that its performance is similar to the old one, with a slightly better precision but substantially lower recall.

Moreover, since the inputs of our model are assembled sequences, its application scenarios may be limited, and it cannot work on short reads directly unless heavy computational pre-processing steps are done. This should be considered as one limitation of our framework. Extending HMD-ARG to classify the metagenomics short reads without assembling is a very promising research direction, which is desirable in many application scenarios. In the future, we will combine our framework with the newest sequencing technology, such as the Nanopore sequencing [46–50], to develop a pipeline that works on the short reads level or even the sequencing signal level.

We believe that HMD-ARG can serve as a powerful tool to alleviate the global threat of the rising abundance and diversity of antibiotic resistant genes. In the future, we will incorporate other dimensions of information, such as 3D structural information [39] and SNPs, into our framework to further improve our method’s performance and extend the application scenarios.

## Supplementary Information

**Additional file 1: Figure S1**: ROC curve comparison. For ARG/Non-ARG prediction, we set different thresholds for the last layer of the HMD-ARG model and different parameters for DeepARG, drawing the above ROC curves. The figure suggests that HMD-ARG is more robust than DeepARG. **Figure S2**: Human intestinal microbiota prediction. After removing overlaps between HMD-ARG training data and the Mustard database, we trained a new model and applied the model to this human gut dataset. In the figure, we show the number of correctly predicted ARGs, by HMD-ARG and DeepARG, across different classes in the Mustard dataset. Compared to DeepARG, HMD-ARG can recover much more correct ARGs in more diverse classes, which suggests the robustness and the sensitivity of the proposed method. **Figure S3**: Growth curves of *E. coli* host with the expression of the predicted novel ARGs that inactivate antibiotics in the presence of antibiotics. a. The growth curve of *E. coli* under the presence of Ampicillins (50 μg/ml) b. The same to a, while removes the AXX01_04100, which is a predicted novel ARGs shares high similarity compared with known ones. c. The growth curve of *E. coli* under the presence of Carbenicillin (10 μg/ml) d. The same to c, while removes the AXX01_04100, which is a predicted novel ARG that shares high similarity compared with known ones. **Figure S4**: Correlation between saliency map and PSSM. a. For each position in a sequence, which is shown as the columns, we mutated the amino acid to the other amino acids, which are shown as the rows, and fed the mutated sequence to HMD-ARG model, determining the probability of the sequence being an ARG and filling in the value into the corresponding position in the saliency map with the probability. The figure shows the averaged saliency map of those sequences, which can be aligned to AFB78806. b. The figure shows the position-specific scoring matrix (PSSM) of AFB78806, which indicates the evolutionary information of that ARG. We can find a clear correlation between a) and b), especially for row c (horizontal rectangle) and column 226 (vertical rectangle). This correlation suggests that although we only used the protein sequence as input, without resorting to sequence alignment, HMD-ARG can capture the evolutional information of ARG sequences, which demonstrates the effectiveness of the proposed method. **Figure S5**: The saliency map built with PSSM. The PSSM predicted conserved sites and the corresponding sequence logo from 319 to 393 in AXX01_04100. **Table S1**: The list of ARGs predicted in the study and validated using heterogenous expression in *E. coli* host. **Table S2**: Primers used for constructing overexpression plasmids.

## Data Availability

The database and the software server are available at http://www.cbrc.kaust.edu.sa/HMDARG/.
