# Supplementary material for: HMD-ARG: hierarchical multi-task deep learning for annotating antibiotic resistance genes
Source: Microbiome. 2021 Feb 8;9:40. doi: 10.1186/s40168-021-01002-3 (PMC7871585; doi:10.1186/s40168-021-01002-3)
Supplement: Supplementary file 2 — Additional file 1: Figure S1: ROC curve comparison. For ARG/Non-ARG prediction, we set different thresholds for the last layer of the HMD-ARG model and different parameters for DeepARG, drawing the above ROC curves. The figure suggests that HMD-ARG is more robust than DeepARG. Figure S2: Human intestinal microbiota prediction. After removing overlaps between HMD-ARG training data and the Mustard database, we trained a new model and applied the model to this human gut dataset. In the figure, we show the number of correctly predicted ARGs, by HMD-ARG and DeepARG, across different classes in the Mustard dataset. Compared to DeepARG, HMD-ARG can recover much more correct ARGs in more diverse classes, which suggests the robustness and the sensitivity of the proposed method. Figure S3: Growth curves of E. coli host with the expression of the predicted novel ARGs that inactivate antibiotics in the presence of antibiotics. a. The growth curve of E. coli under the presence of Ampicillins (50 μg/ml) b. The same to a, while removes the AXX01_04100, which is a predicted novel ARGs shares high similarity compared with known ones. c. The growth curve of E. coli under the presence of Carbenicillin (10 μg/ml) d. The same to c, while removes the AXX01_04100, which is a predicted novel ARG that shares high similarity compared with known ones. Figure S4: Correlation between saliency map and PSSM. a. For each position in a sequence, which is shown as the columns, we mutated the amino acid to the other amino acids, which are shown as the rows, and fed the mutated sequence to HMD-ARG model, determining the probability of the sequence being an ARG and filling in the value into the corresponding position in the saliency map with the probability. The figure shows the averaged saliency map of those sequences, which can be aligned to AFB78806. b. The figure shows the position-specific scoring matrix (PSSM) of AFB78806, which indicates the evolutionary information of that ARG. We can find a cl [file 40168_2021_1002_MOESM2_ESM.docx]

**Supplemental Materials for**

**HMD-ARG: Hierarchical Multi-task Deep learning for Annotating Antibiotic Resistance Genes**

**
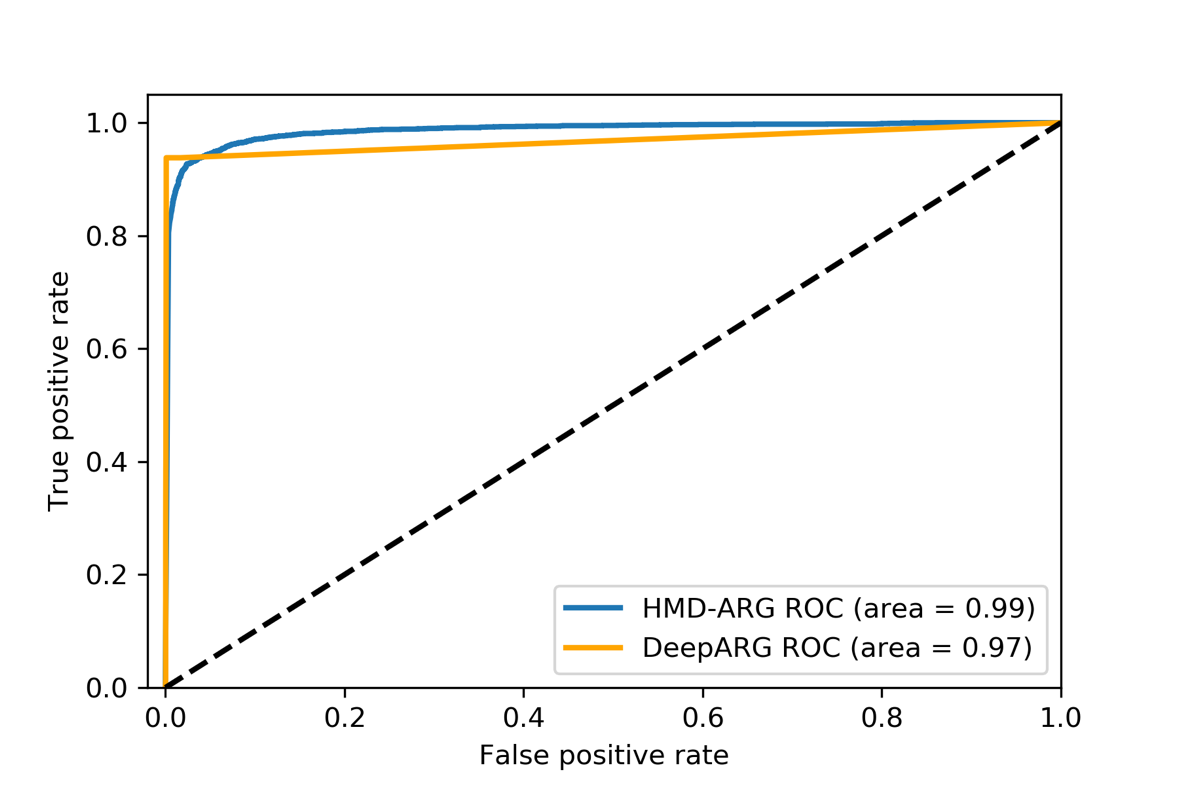
**

**Fig. S1: ROC curve comparison.** For ARG/Non-ARG prediction, we set different thresholds for the last layer of the HMD-ARG model and different parameters for DeepARG, drawing the above ROC curves. The figure suggests that HMD-ARG is more robust than DeepARG.

**
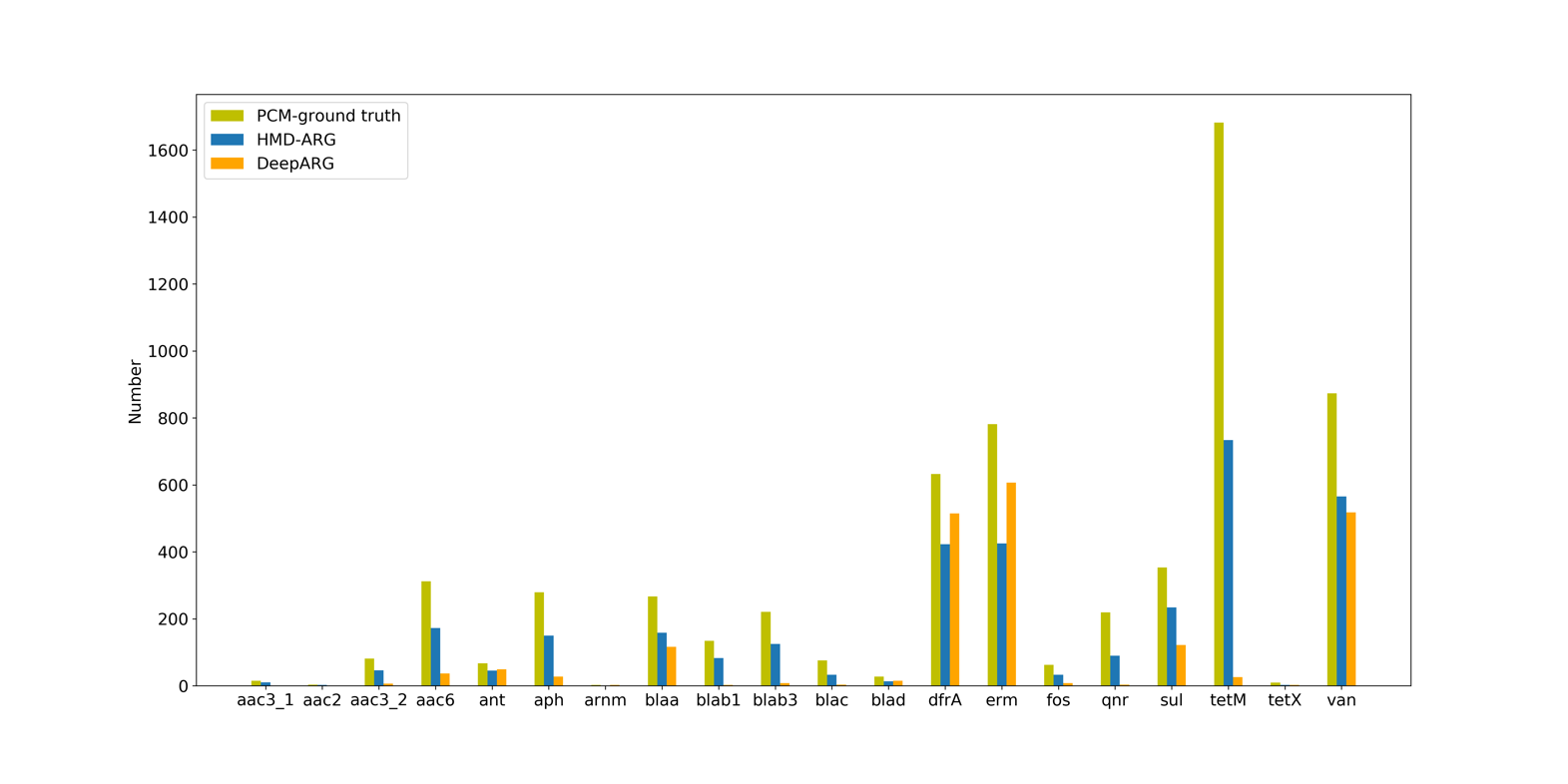
**

**Fig. S2: Human intestinal microbiota prediction.** After removing overlaps between HMD-ARG training data and the Mustard database, we trained a new model and applied the model to this human gut dataset. In the figure, we show the number of correctly predicted ARGs, by HMD-ARG and DeepARG, across different classes in the Mustard dataset. Compared to DeepARG, HMD-ARG can recover much more correct ARGs in more diverse classes, which suggests the robustness and the sensitivity of the proposed method.


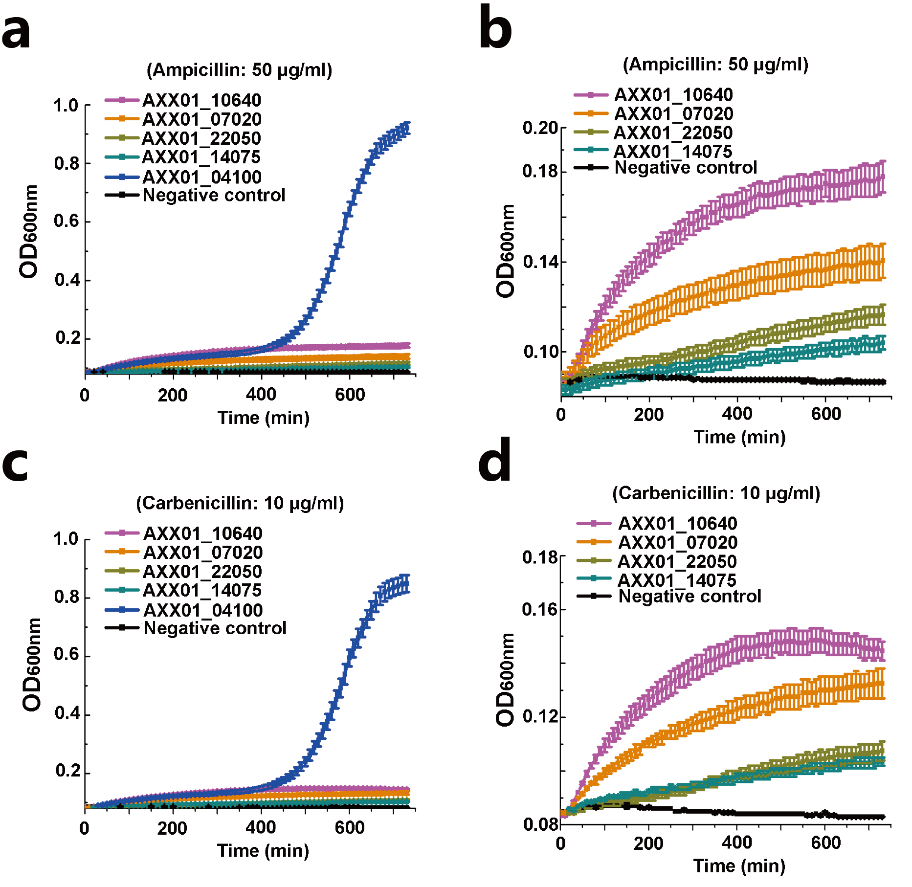


**Fig. S3: Growth curves of *E. coli* host with the expression of the predicted novel ARGs that inactivate antibiotics in the presence of antibiotics.** **a.** The growth curve of *E. coli* under the presence of Ampicillins (50 μg/ml) **b.** The same to a, while removes the AXX01_04100, which is a predicted novel ARGs shares high similarity compared with known ones. **c.** The growth curve of *E. coli* under the presence of Carbenicillin (10 μg/ml) **d.** The same to c, while removes the AXX01_04100, which is a predicted novel ARG that shares high similarity compared with known ones.

**
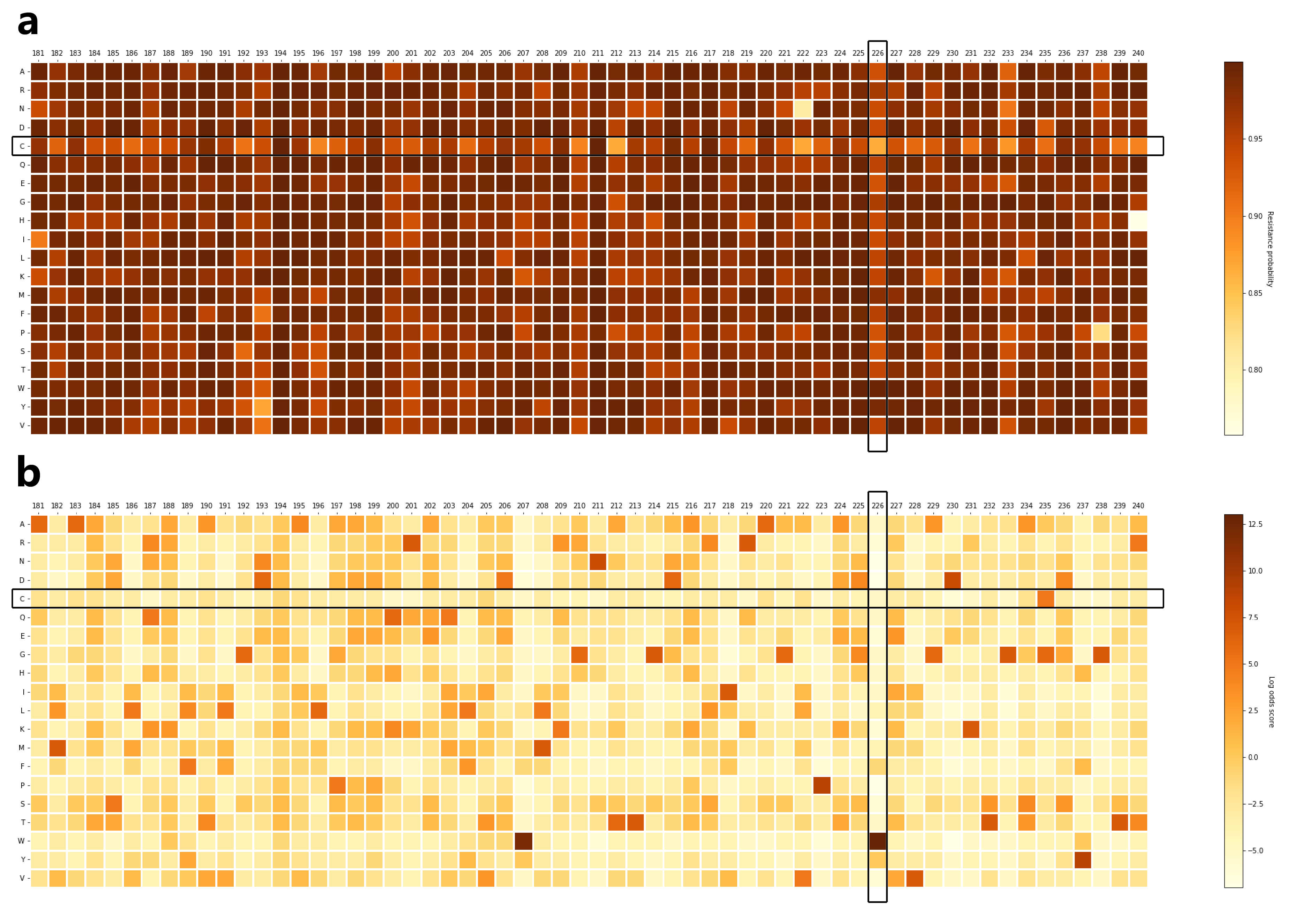
**

**Fig. S4: Correlation between saliency map and PSSM. a.** For each position in a sequence, which is shown as the columns, we mutated the amino acid to the other amino acids, which are shown as the rows, and fed the mutated sequence to HMD-ARG model, determining the probability of the sequence being an ARG and filling in the value into the corresponding position in the saliency map with the probability. The figure shows the averaged saliency map of those sequences, which can be aligned to AFB78806. **b.** The figure shows the position-specific scoring matrix (PSSM) of AFB78806, which indicates the evolutionary information of that ARG. We can find a clear correlation between a) and b), especially for row c (horizontal rectangle) and column 226 (vertical rectangle). This correlation suggests that although we only used the protein sequence as input, without resorting to sequence alignment, HMD-ARG can capture the evolutional information of ARG sequences, which demonstrates the effectiveness of the proposed method.


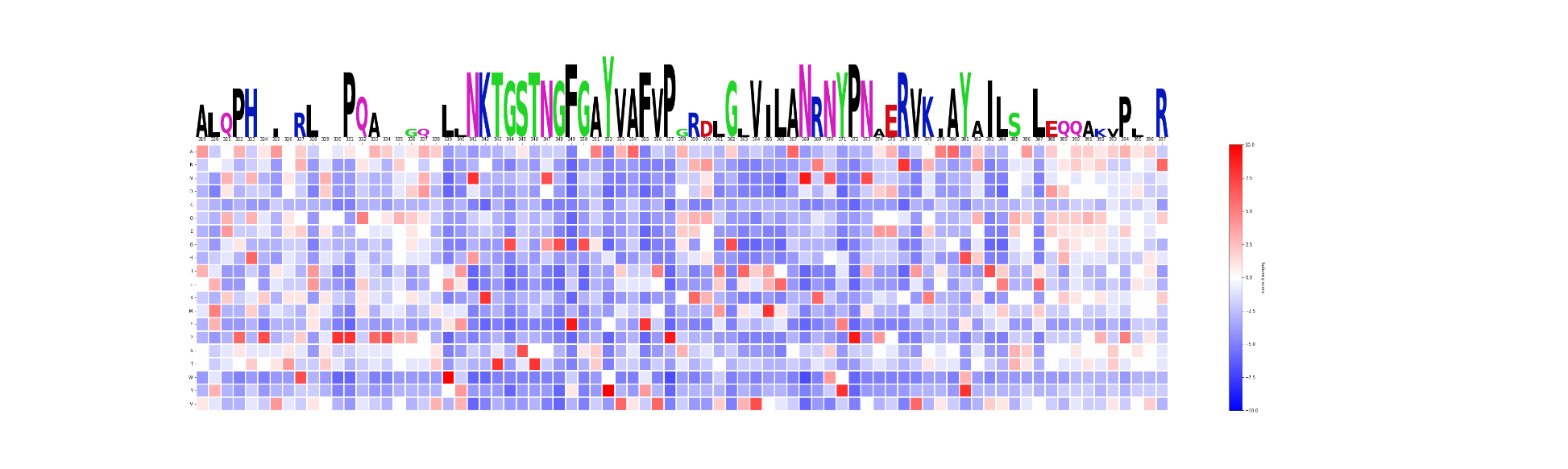


**Fig. S5: The saliency map built with PSSM.** The PSSM predicted conserved sites and the corresponding sequence logo from 319 to 393 in AXX01_04100.

**Database construction**

We collected and cleaned antibiotic resistance genes from seven published ARG database. They are Comprehensive Antibiotic Resistance Database (CARD), AMRFinder, ResFinder, Antibiotic Resistance Gene-ANNOTation (Arg-ANNOT), DeepARG, MEGARes, Resfams. Then we assigned those ARGs with three kinds of annotations: the antibiotic class they confer to, the mechanism of antibiotic resistance, and transferable ability. For our main goal, the antibiotic class they confer to, we adopted labels from their source databases and manually aligned the label with DeepARG. For instance, the resistance gene labeled with rifampin or rifamycins can be considered as the same antibiotic group; we labeled both as rifamycins.

As for resistance mechanism annotation, we used the ontology system from CARD and assigned mechanism labels to ARGs using BLASTP and best-hit strategy with a cut-off score of 1e-20. There are 1994 sequences in our database that missed tags under this condition, so we manually checked the original publications and assigned labels accordingly.

For gene mobility(transferability), we used AMRFinder, the up-to-date acquired ARGs database for label annotation. We used the command line tool offered by AMRFinder, which includes both sequence alignment method and HMM profiles for discriminating whether the ARG is an acquired one.

**Evaluation criteria**

To comprehensively benchmark the classification performance of the proposed method, we performed cross-fold validation experiments on all the three levels and compared HMD-ARG with the state-of-the-art methods, including CARD, DeepARG, and CNN-BLPred. The predictions were evaluated with accuracy, macro precision, macro recall and macro F1 score, which are defined using true positives (TP), true negatives (TN), false positives (FP) and false negatives (FN). We also made use of the receiver-operating characteristic (ROC) curve, which operates with a threshold and presents how TP varies along with FP. In addition, we reported the area under the ROC curve (AUROC) to evaluate model performance in a quantitative and comparable setting.

Below is the formal definitions of these criteria:

$accuracy\left( y, \hat{y} \right)=\frac{1}{n_{samples}}\sum_{i=0}^{n_{samples}-1} 1(y_{i}= \hat{y}_{i})$,

$Macro-precision\left( y, \hat{y} \right)=\frac{1}{\left| M \right|}\sum_{l\in M} precision(y_{l}, \hat{y}_{l})$,

$Macro-recall\left( y, \hat{y} \right)=\frac{1}{\left| M \right|}\sum_{l\in M} recall\left( y_{l}, \hat{y}_{l} \right)$,

$Macro-F1 score\left( y, \hat{y} \right)=\frac{1}{\left| M \right|}\sum_{l\in M} F1 score(y_{l}, \hat{y}_{l})$,

where $n_{samples}$ is the total number of samples; $1(y_{i}= \hat{y}_{i})$ is the indicator function of the predicated label as the true label; $y_{i}$ is the ground truth label for sample $i$; $\hat{y}_{i}$ is the predicted label for sample $i$; $M$ is the set of labels; $y_{l}$ is the subset of y with label $l$; and $\hat{y}_{l}$ is the predicted label for the subset.

**Protocols of other methods:**

**DeepARG:**

Test data: our cross-validation data

Version: 2019-08

Commands:

deeparg predict --model LS --type prot –input path_to_input --out path_to_output --evalue 1 --iden 60

**CARD:**

Test data: our cross-validation data

Version: 2019-08

Commands:

blastp -db card -query path_to_input -out path_to_output

**AMRPlusPlus:**

Test data: AMRPlusPlus can only accept paired fastq sequences, one forward and one backward. So 150 bp reads were simulated with a mean insert size of 200 bp and standard deviation of 10 bp at 2x coverage over the ARGs in our cross-validation data with a HiSeq Illumina error profile using ART.

Version: 2.0

Commands:

nextflow run main_AmrPlusPlus_v2.nf -profile singularity --reads path_to_input_{1,2}.fq --output path_to_output

**Meta-MARC**

Test data: We back translated ARGs in our cross-validation data

Version: 2017-03

Commands:

mmarc -i path_to_input -o path_to_output -l 1 -t 50

**Model hype-parameters**

The hyper-parameters in the HMD-ARG model include model architecture, the kernel size and the number of kernels in the convolutional layer, the pooling kernel size of the max-pooling layer, the dropout rate, the optimizer algorithm, and learning rate. Since we focus on the classification of all three tasks, we used cross-entropy as the loss function. Specifically, our level 1 model performs multi-task learning for antibiotic family, mechanism of antibiotic resistance, and gene mobility simultaneously with a weighted sum loss function on the three tasks: $L_{multi-task}=\alpha*L_{drug}+\beta*L_{mechanism}+\gamma*L_{mobility}$.

The hyper-parameters are summarized as follows:

| Architecture | layer | kernel size | kernel number |
| --- | --- | --- | --- |
|  | conv1 | 40*4 | 32 |
|  | max-pooling1 | 5*2 | NA |
|  | conv2 | 30*4 | 64 |
|  | conv3 | 30*4 | 128 |
|  | max-pooling2 | 5*2 | NA |
|  | conv4 | 20*3 | 256 |
|  | conv5 | 20*3 | 256 |
|  | max-pooling3 | 4*1 | NA |
|  | conv6 | 20*3 | 256 |
|  | max-pooling4 | 2*1 | NA |
|  | fc1 | 12288*1024 | NA |
|  | fc2 | 1024*1024 | NA |
|  | drug target task | 1024*15 | NA |
|  | mechanism task | 1024*6 | NA |
|  | gene source task | 1024*2 | NA |
|  | stride | 1 | NA |
| Optimizer | Adam | | |
| Learning rate | 1e-4 | | |
| Dropout rate | 0.9 | | |
| Activation function | ReLU | | |
| Multi-task learning hyper-paramters | $\alpha=1$ | $\beta=0.2$ | $\gamma=0.2$ |

**Table S1: The list of ARGs predicted in the study and validated using heterogenous expression in *E. coli* host.**

| Locus tag in genome | Antibiotic class predicted | Blast hits in arg7db | Blast identity against hit in arg7db (%) | Annotations from genome |
| --- | --- | --- | --- | --- |
| AXX01_10640 | beta_lactam | WP_000465449.1\|FEATURES\|argannot\|beta_lactam\|BL3_l | 28.947 | MBL fold metallo-hydrolase |
| AXX01_07020 | beta_lactam | KF629893.1_12522\|FEATURES\|farme\|beta_lactam\|BL3_l | 40.558 | MBL fold metallo-hydrolase |
| AXX01_22050 | beta_lactam | ACI02045.1\|FEATURES\|farme\|beta_lactam\|PEDO-2 | 34.804 | MBL fold metallo-hydrolase |
| AXX01_04100 | beta_lactam | YP_789012\|FEATURES\|ARDB_deepARG\|beta_lactam\|PDC-9 | 100 | class C beta-lactamase |
| AXX01_14075 | beta_lactam | AIA11032.1\|FEATURES\|farme\|beta_lactam\|ampC | 30.732 | serine hydrolase |
| AXX01_04055 | aminoglycoside | A0A0H2ZEU7\|FEATURES\|UNIPROT_deepARG\|aminoglycoside\|aph | 100 | APH(3') family aminoglycoside O-phosphotransferase |
| AXX01_04925 | aminoglycoside | AIA17659.1\|FEATURES\|farme\|aminoglycoside\|SG34_20990 | 47.674 | GNAT family N-acetyltransferase |
| AXX01_26535 | aminoglycoside | AIA14994.1\|FEATURES\|farme\|chloramphenicol\|catQ | 43.827 | GNAT family N-acetyltransferase |

| **Table S2: Primers used for constructing overexpression plasmids.** | |
| --- | --- |
| **Primer ID** | **Primer sequence (5’-3’)** |
| pET-AXX01_10640-F | CGGGATCCATGTTGAAACCCGACATCAC |
| pET-AXX01_10640-R | CCGCTCGAGTCAGAACAGATCCAGCGGG |
| pET-AXX01_07020-F | CGGGATCCATGCGGTTCGCGGTTCTGGG |
| pET-AXX01_07020-R | CCGCTCGAGCTAGGCGATCTCGCGCCAAT |
| pET-AXX01_22050-F | CGGGATCCATGTCGACATCCCCCGCGCT |
| pET-AXX01_22050-R | CCGCTCGAGTCAGCCCCTGACGAAGGGAT |
| pET-AXX01_04100-F | CGGGATCCATGCGCGATACCAGATTCC |
| pET-AXX01_04100-R | CCGCTCGAGTCAGCGCTTCAGCGGCACCT |
| pET-AXX01_14075-F | CGGGATCCATGTCAGGTTTCGAACGAG |
| pET-AXX01_14075-R | CCGCTCGAGTCAGCGCACCTCCGCGGAAC |
| pET-AXX01_04055-F | CGGGATCCATGCATGATGCAGCCACCTC |
| pET-AXX01_04055-R | CCGCTCGAGCTAGAAGAACTCGTCCAATAG |
| pET-AXX01_04925-F | CGGGATCCATGAACGCGAACCTTCCCCC |
| pET-AXX01_04925-R | CCGCTCGAGTCACCGCTCCGCCCAGCGG |
| pET-AXX01_26535-F | CGGGATCCATGAGCGCTTCGATCCGCG |
| pET-AXX01_26535-R | CCGCTCGAGTCAGGGCGCGCTGCGCGTC |
